# Investigation and manipulation of metabolically active methanogen community composition during rumen development in black goats

**DOI:** 10.1038/s41598-017-00500-5

**Published:** 2017-03-24

**Authors:** Zuo Wang, Chijioke O. Elekwachi, Jinzhen Jiao, Min Wang, Shaoxun Tang, Chuanshe Zhou, Zhiliang Tan, Robert J. Forster

**Affiliations:** 1 Key Laboratory for Agro-Ecological Processes in Subtropical Region, Hunan Research Center of Livestock & Poultry Sciences, South-Central Experimental Station of Animal Nutrition and Feed Science in Ministry of Agriculture, Institute of Subtropical Agriculture, Chinese Academy of Sciences, Changsha, Hunan 410125 China; 2 University of Chinese Academy of Sciences, Beijing, 100049 China; 3 Lethbridge Research and Development Centre, Agriculture and Agri-Food Canada, Lethbridge, AB T1J 4B1 Canada

## Abstract

This study was performed to investigate the initial colonization of metabolically active methanogens and subsequent changes in four fractions: the rumen solid-phase (RS), liquid-phase (RL), protozoa-associated (RP), and epithelium-associated (RE) from 1 to 60 d after birth, and manipulate methanogen community by early weaning on 40 d and supplementing rhubarb from 40 to 60 d in black goats. The RNA-based real-time quantitative PCR and 16S rRNA amplicon sequencing were employed to indicate the metabolically active methanogens. Results showed that active methanogens colonized in RL and RE on 1 d after birth. RP and RE contained the highest and lowest density of methanogens, respectively. *Methanobrevibacter*, Candidatus *Methanomethylophilus*, and *Methanosphaera* were the top three genera. The methanogen communities before weaning differed from those post weaning and the structure of the methanogen community in RE was distinct from those in the other three fractions. The discrepancies in the distribution of methanogens across four fractions, and various fluctuations in abundances among four fractions according to age were observed. The addition of rhubarb significantly (P < 0.05) reduced the abundances of *Methanimicrococcus* spp. in four fractions on 50 d, but did not change the methanogen community composition on 60 d.

## Introduction

The rumen accommodates various prokaryotic (bacteria and archaea) and eukaryotic (protozoa and fungi) microorganisms that symbiotically degrade and ferment the feed ingested by the host ruminant^1^. The ruminal microbiota is characterized by its high population density, wide diversity, and complexity of interactions, and it is suggested that the abundance of various microbial genotypes within the rumen can be related to host feed efficiency and diet^2, 3^. In young ruminants and during rumen development, ingested microbes colonize and establish in a defined and progressive sequence^4–6^. During the last few decades, intensive efforts have been taken to explore the relationship between microbial colonization and rumen development using different methods^5, 7–9^, as the composition of ruminal microorganisms directly influences the digestive and metabolic performance of the host. The developing rumen in the newborn ruminants offers a unique chance to manipulate the complex commensal microbiota^10^.

It has been found that the early dietary experiences of the animal can have a greater and more lasting effect than those occurring later in life^11^. This would possibly allow the manipulation of the rumen microbial community at the early period of rumen development, i.e., microbial programming^12^. Li *et al*.^13^ reported that the microbiome in the developing rumen of 14 days old calves was responsive to dietary modifications as well as physiological changes in the host. Further studies^4, 14^ implied that it would be possible to promote the establishment of different microbial populations in the rumen of the young animal by controlling feed management in early life. However, insight into the development of the rumen and its microbiome, the method (e.g., the alteration of diets and the inoculation of specific additives) and timing to manipulate the ruminal microbiome in early life is still lacking.

Among the vast and diverse ruminal microbiota, the methanogens constitute the majority of the domain Archaea that can account for up to 3–4% of the entire microbial population^15, 16^. Methanogens utilize H_2_ as the energy source to reduce CO_2_ to CH_4_, which is essential to prevent the accumulation of reducing equivalents and the overall inhibition of ruminal fermentation^17^. Methane (CH_4_) produced in the rumen causes the loss of about 2–12% of the gross energy intake of the host, and is a potent greenhouse gas with a global warming potential which is 25 times that of CO_2_^18^. Therefore, increased knowledge of the microbiology of methanogenesis and evaluating approaches to regulate the ruminal methanogenic population are vital to the mitigation of enteric greenhouse gas to provide increases in production efficiency in the livestock industry^19^. In addition, most of the studies on ruminal methanogens were either focused on the solid-associated and/or liquid-associated^9, 20–22^, or the protozoa-associated fractions^23, 24^. Hence, it would be of great significance to conduct research targeting the evolution of the methanogen population across all the four fractions, i.e., the rumen solid-phase (RS), liquid-phase (RL), protozoa-associated (RP), and epithelium-associated (RE).

Rhubarb (*Rheum* spp.) is commonly used as a herb in traditional Chinese medicine that contains anti-microbial ingredients, such as such as emodin, aloe-emodin and rhein^25^, and may be a potential CH_4_ mitigation agent. Previous *in vitro* and *in vivo* investigations found that rhubarb could inhibit ruminal methanognesis, and alter rumen fermentation through propionate production^26, 27^. However, these studies were aimed at regulating rumen fermentation in mature ruminants. To our current knowledge, there is still in lack of evidence concerning the effects of rhubarb on the rumen methanogen community in early life during rumen development.

Until recently, the overwhelming majority of the investigations on the rumen microbiota employing 16S rRNA gene sequencing have been performed based on DNA-derived amplicons^8, 28–30^, which could reflect the comprehensive diversity of all the living and inactive microorganisms. However DNA-based studies do not reflect the potential biological activity of the rumen microbial community in real time^31, 32^. In contrast, RNA-based techniques could help obtain insights into the metabolic state of microbes and thus could be used to indicate the most active rumen microorganisms and their metabolic potential^33, 34^.

In the present study, we aimed to deepen the understanding of methanogen colonization during rumen development in order to facilitate the manipulation of the rumen microbiome and fermentation in early life using theoretical foundations. We used RNA-based real-time quantitative PCR (qPCR) and 16S rRNA amplicon sequencing to investigate the initial colonization and diversity of the metabolically active methanogens in four fractions (i.e., RS, RL, RP, and RE) and the subsequent evolution from 1 to 60 d after birth. The influences of early weaning on 40 d and the supplementation of rhubarb from 40 to 60 d on the community of methanogens in the rumen were also examined in this study.

## Results

### Quantitation of rumen methanogens in four fractions during rumen development and with supplementation of rhubarb

The methanogen copy numbers were significantly (*P* < 0. 01) different amongst fractions, and an interaction (*P* < 0.01) between fraction and age was observed (Table 1). The copy number of methanogens in the RS fraction was generally higher compared to the other three fractions, and it rose quadratically (*P* < 0.01) and had become steady as the goats aged past 38 d. For the RL fraction, the methanogen copy number was numerically or significantly (*P* < 0.05) less compared to the RP fraction, but higher than that of the RE fraction. A quadratic (*P* < 0.05) increase of copy number in RL fraction in response to age was noted, and the copy number stabilized from 10 d. In the RP fraction, the copy number of methanogens generally stayed stable and was always significantly (*P* < 0.05) greater than that of the RE fraction except at 38 d. In general, the methanogen archaea copy number in the RE fraction was the lowest compared to RS, RL and RP, and a linear (*P* < 0.01) rise was observed reaching a peak at 50 d. Statistical analysis indicated that although the methanogen copy numbers in RL and RE fractions in the rhubarb group were numerically lower compared to the control, there were no significant (*P* < 0.05) differences in the estimated copy numbers of methanogens between the two diet treatments after weaning (see Supplementary Table S1).

**Table 1:** Change of methanogen copy number (Log_10 _copies/μL cDNA) in four fractions during rumen development^a,b^.

Fraction	Age (d)^1^							SEM^2^	Significance (P<)^3^		
	1	10	20	38	41	50	60		Fraction	Age	Fraction × Age
RS	—^4^	—	6.838^bA^	8.809^aA^	8.930^aA^	8.989^aA^	8.458^aA^	0.5281	<0.01	Q (<0.01)	<0.01
RL	5.280^b^	6.355^aA^	6.923^aA^	7.093^aB^	6.387^aBC^	6.903^aBC^	6.013^abB^			Q (<0.05)	
RP	—	—	7.588^aA^	6.062^bBC^	7.367^abB^	7.484^aB^	7.495^aA^			NS	
RE	4.963^b^	4.934^bB^	5.154^abB^	5.403^abC^	5.285^abC^	6.100^aC^	5.881^abB^			L (<0.01)	

Means within a row for days that do not have a common superscript differ (P < 0.05); ^A–C^Means within a column for fractions that do not have a common superscript differ (P < 0.05). ^1^Samples on 50 d and 60 d were collected from goats fed the control diet; SEM ^2^represents SEM for fraction × age; ^3^NS = not significant (P > 0.05), L = Linear effect of age, Q = Quadratic effect of age; ^4^No data due to the absence of samples.

### Rumen methanogen community composition in four fractions during rumen development and with supplementation of rhubarb

According to the taxonomic assignment, a total of 61 methanogenic genera were identified throughout the four fractions in the rumen of black goats (see Supplementary Fig. S1). Before weaning, *Methanobrevibacter*, Candidatus *Methanomethylophilus*, and *Methanosphaera* were the three most common genera in the four fractions, which in total represented from 89.8% to 98.3% of methanogens across individuals. After weaning, the abundances of *Methanomicrobium* spp. and *Methanimicrococcus* spp. increased dramatically and together accounted for up to 46.8% of methanogens. Moreover, the abundance of the genus *Methanimicrococcus* then decreased from 50 to 60 d and lost its dominance.

In contrast to the control diet group, the genus *Methanimicrococcus* was not among the dominant genera in all the four fractions of the rhubarb treatment on 50 d (see Supplementary Fig. S2). The abundance of *Methanimicrococcus* spp. in the control ranged from 15.4% to 40.1% across individuals, was reduced to as low as 1.4% in the rhubarb treatment. However, there were no noticeable differences in the community composition of methanogens between the two treatments on 60 d.

### Diversity of rumen methanogens in four fractions during rumen development and with rhubarb treatment

**Figure 1:**
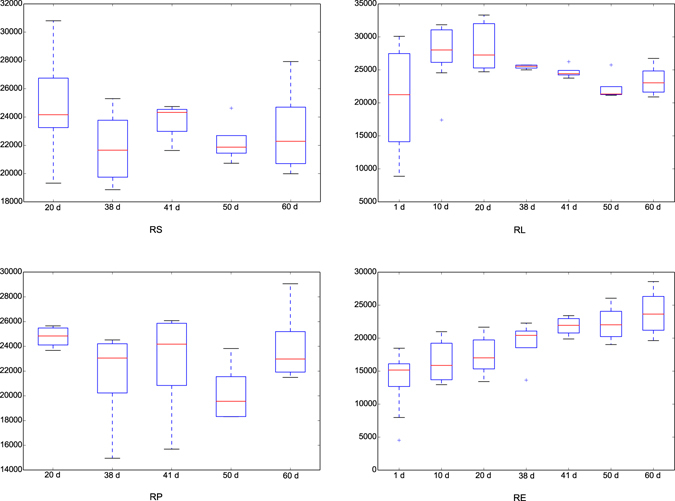
Comparison of Chao 1 index of methanogen communities within different ages for each fraction. The horizontal lines in each box indicate the median values, and the 75^th^ and 25^th^ quartile values are respectively represented by the top and bottom sides of each box.

**Figure 2:**
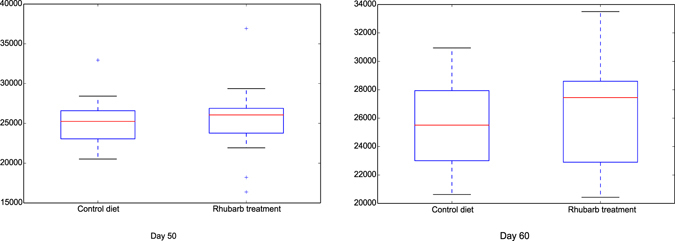
Comparison of Chao 1 index of methanogen communities between control diet treatment and rhubarb supplementation treatment on 50 d and 60 d. The horizontal lines in each box indicate the median values, and the 75^th^ and 25^th^ quartile values are respectively represented by the top and bottom sides of each box.

The methanogen diversities among different days in each fraction were estimated and contrasted using the Chao 1 indice of Alpha diversity (Fig. 1). An age-dependent increment was observed in the fraction of RE, but this pattern was not found in the other three fractions. In addition, the comparison of Chao 1 amongst fractions on different days showed that on 20 d the richness in RE was lower than those of the other three fractions, and on 41 d the Chao 1 value in RS was greater compared to the fractions of RP and RE (see Supplementary Fig. S3). Further, no differences in the Chao 1 index between the control diet and rhubarb treatment were apparent on 50 d or on 60 d (Fig. 2).

**Figure 3:**
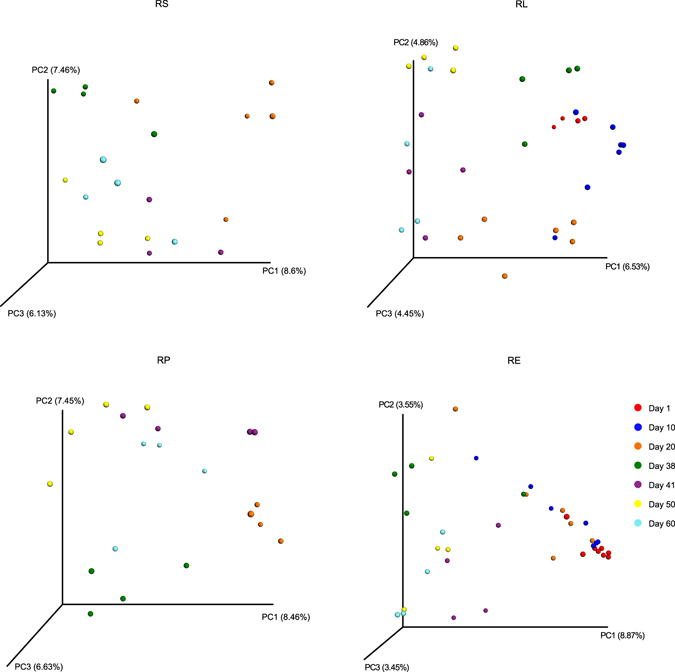
Principal coordinate analysis (PCoA) of methanogen community structure using unweighted Unifrac matrix within different ages for each fraction.

The beta diversities of methanogen communities within different ages for each fraction were calculated and visualized using principal coordinate analysis based on all core OTUs (Fig. 3). In the fraction of RS, the methanogen communities on 20 d and 38 d were relatively close and distinct from those communities after weaning. For RL, the communities before weaning clustered distinctly from those days after weaning. In RP, the methanogen communities of 20 d and 38 d were separate from each other but still different from those communities after weaning. In RE, the methanogen community on 41 d was comparatively isolated from the communities of the other days. Methanogen communities of RE and RL were different from each other on 1 d and 10 d (see Supplementary Fig. S4), and the methanogen community in RE was always isolated from the communities in the other three fractions. At 60 d, the communities of RE and RL clustered separately from each other, while no clear clustering was observed for RS and RP.

**Figure 4:**
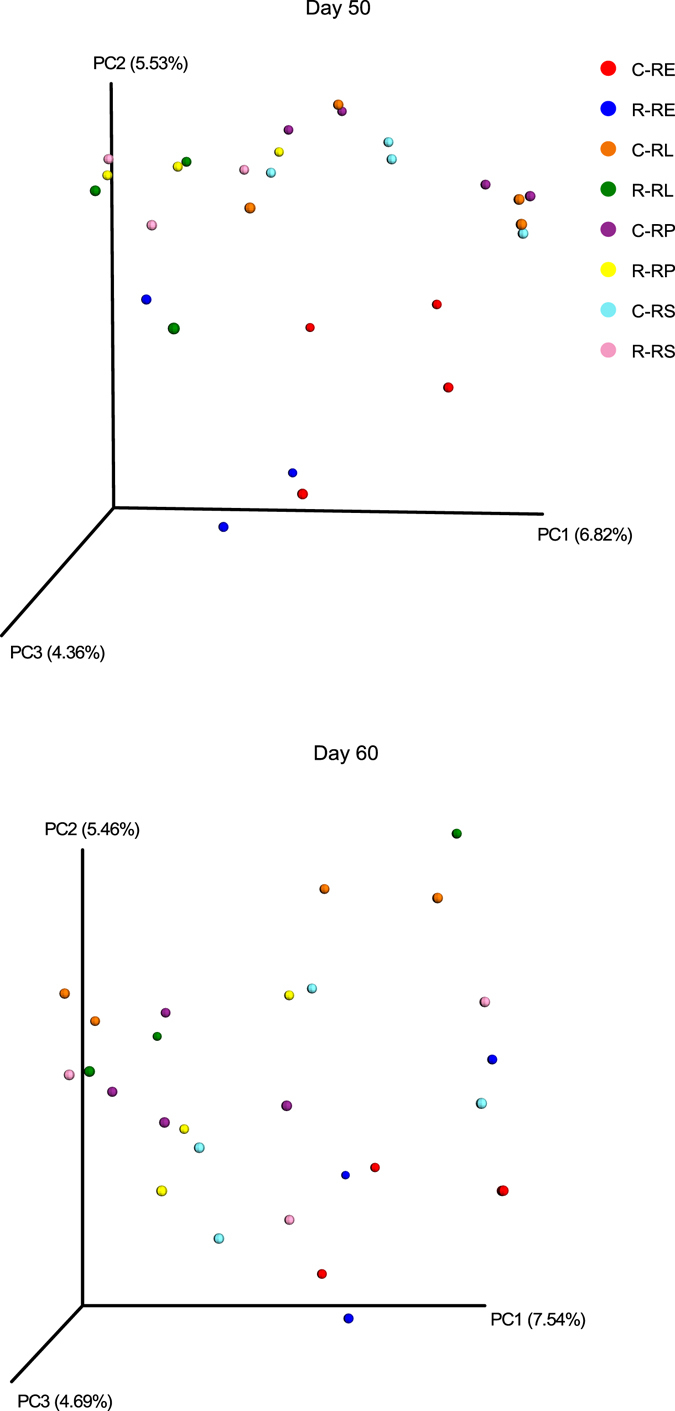
Principal coordinate analysis (PCoA) of methanogen community structure using unweighted Unifrac matrix between two treatments (control diet [C] and rhubarb supplementation [R]) on 50 d and 60 d.

On 50 d, the methanogen community in each fraction of the rhubarb treatment was different from that of the control group (Fig. 4). However, no clear clustering pattern between two treatments was noted on 60 d.

### Relative abundance of methanogens in four fractions during rumen development and with rhubarb treatment

After arcsine transformation and subsequent statistical analysis, all the data was converted back into the original percent relative abundance and is presented in Table 2. Statistical analysis showed that sample fraction significantly affected the relative abundances of all the main genera except the *Methanobacterium*, and a highly significant (P < 0.01) interaction between fraction and age was noted on the relative abundances of *Methanobrevibacter* spp., Candidatus *Methanomethylophilus* spp., and *Methanosphaera* spp. For the genus *Methanobrevibacter*, its abundance in RP was the highest when compared with the other three fractions, and it reached a maximum abundance in all fractions on 38 d and then dropped dramatically after weaning. In addition, cubic increases of the abundance of *Methanobrevibacter* spp. in RL (*P* < 0.01) and RP (*P* < 0.05) were observed, respectively. The abundance of Candidatus *Methanomethylophilus* spp. in RP was always the lowest compared to other fractions until 50 d, and the abundances in RS and RE became lower than RP and RL on 60 d. Age exerted quadratic effects on the abundance of Candidatus *Methanomethylophilus* spp. in RS (*P* < 0.01), RP (*P* < 0.01), and RE (*P* < 0.05), while a cubic (P < 0.01) effect of age was noted in RL. The relative abundance of *Methanosphaera* spp. was the lowest in RL compared to the other three fractions from 20 to 60 d. The abundances in all fractions were significantly (P < 0.05) reduced on 41 d when compared with those on 38 d. Further, a cubic (*P* < 0.05) effect of the abundances in both RS and RL was noted, while the abundances in RP (*P* < 0.05) and RE (*P* < 0.01) rose quadratically with age. For the genus *Methanomicrobium*, its abundance in RP was generally the least compared to other three fractions, and increasing linear effects of age in RL (*P* < 0.01), RP (*P* < 0.05), and RS (*P* < 0.01) were respectively noted. The relative abundance of *Methanobacterium* spp. was much less than the other methanogenic genera, and age quadratically (P < 0.05) affected it in the fraction of RL. It was found that the abundance of *Methanimicrococcus* spp. in the four fractions all rose cubically (*P* < 0.01) with age. Significant (*P* < 0.05) increases were observed in four fractions after weaning on 41 d and 50 d, however followed by significant (*P* < 0.05) decreases on 60 d.

**Table 2:** Change of relative abundance (%) of active methanogenic genera in four fractions during rumen development^a–e^.

Fraction	Age (d)^1^							SEM^2^	Significance (P<)^3^		
	1	10	20	38	41	50	60		Fraction	Age	Fraction × Age
*Methanobrevibacter*											
RS	—^4^	—	34.77^bBC^	61.15^aB^	23.23^bB^	36.89^b^	36.95^bA^	0.682	<0.01	NS	<0.01
RL	45.21^b^	28.70^c^	25.08^cdC^	62.22^aB^	11.95^dB^	31.40^c^	13.93^dB^			C (<0.01)	
RP	—	—	65.78^bA^	81.65^aA^	59.77^bA^	40.17^c^	35.67^cA^			C (<0.05)	
RE	42.20^a^	38.07^a^	38.43^aB^	47.92^aB^	20.73^bB^	32.48^ab^	31.52^abA^			NS	
Candidatus *Methanomethylophilus*											
RS	—	—	55.04^aA^	13.29^cAB^	35.04^abA^	13.43^c^	28.57^bB^	0.744	<0.01	Q (<0.01)	<0.01
RL	38.50^bc^	59.27^a^	65.66^aA^	26.62^cdA^	51.93^abA^	14.77^d^	53.15^abA^			C (<0.01)	
RP	—	—	31.12^abB^	6.10^cB^	14.25^bcB^	4.62^c^	47.03^aA^			Q (<0.01)	
RE	37.52^ab^	52.65^a^	49.96^aA^	26.47^bcA^	33.54^abA^	23.45^bc^	16.98^cB^			Q (<0.05)	
*Methanosphaera*											
RS	—	—	1.42^d^	23.44^aA^	6.62^cAB^	15.23^bAB^	16.26^bB^	0.137	<0.01	C (<0.05)	<0.01
RL	9.19^a^	8.30^a^	0.43^c^	7.79^aB^	1.27^bcC^	5.10^abC^	3.22^abcC^			C (<0.05)	
RP	—	—	0.86^c^	10.54^aB^	4.22^bB^	12.05^aB^	6.84^abC^			Q (<0.05)	
RE	10.03^d^	7.04^de^	1.40^e^	23.75^abA^	10.08^cdA^	20.32^bcA^	35.39^aA^			Q (<0.01)	
*Methanomicrobium*											
RS	—	—	5.32^bc^	0.23^c^	23.10^aA^	13.17^abAB^	14.75^abAB^	0.784	<0.01	NS	NS
RL	3.59^bc^	0.06^c^	0.59^c^	0.37^c^	27.37^aA^	17.13^abA^	27.55^abA^			L (<0.01)	
RP	—	—	0.08	0.11	3.60^B^	1.83^B^	2.82^B^			L (<0.05)	
RE	7.44^bc^	0.14^c^	4.46^bc^	0.44^c^	27.02^aA^	6.46^bcAB^	14.35^abAB^			L (<0.01)	
*Methanobacterium*											
RS	—	—	0.02	0.03	0.02^AB^	0.02	0.03^A^	0.001	NS	NS	NS
RL	0.01	0.01	0.01	0.03	0.01^AB^	0.01	0.01^B^			Q (<0.05)	
RP	—	—	0.01	0.02	0.02^A^	0.01	0.01^B^			NS	
RE	0.02^b^	0.04^a^	0.02^ab^	0.02^ab^	0.01^abB^	0.02^ab^	0.02^abAB^			NS	
*Methanimicrococcus*											
RS	—	—	2.61^b^	0.44^b^	11.17^a^	20.22^aBC^	2.57^b^	0.488	<0.05	C (<0.01)	NS
RL	0.37^c^	0.02^c^	3.15^c^	0.02^c^	6.27^b^	29.65^aAB^	1.24^c^			C (<0.01)	
RP	—	—	1.49^cd^	0.13^d^	17.22^b^	40.09^aA^	6.96^c^			C (<0.01)	
RE	1.40^b^	0.23^b^	4.05^b^	0.29^b^	7.91^a^	15.20^aC^	0.85^b^			C (<0.01)	

Means within a row for days that do not have a common superscript differ (P < 0.05);^A–C^Means within a column for fractions that do not have a common superscript differ (P < 0.05). ^1^Samples on 50 d and 60 d were collected from goats fed the control diet; SEM ^2^represents SEM for fraction × age; ^3^NS = not significant (P > 0.05), L = Linear effect of age, Q = Quadratic effect of age, C = Cubic effect of age; ^4^No data due to the absence of samples.

The supplementation of rhubarb significantly influenced the relative abundances of *Methanobrevibacter* spp. (P < 0.05) and *Methanimicrococcus* spp. (*P* < 0.01) in all the four fractions, and the interaction between diet and age was noted only for *Methanimicrococcus* spp. (*P* < 0.01) (Table 3). In the rhubarb treatment, the abundances of the genus *Methanobrevibacter* in RL was significantly (*P* < 0.05) higher, while the abundances in RS, RP, and RE were numerically greater than those of the control diet group on 50 d. When compared with the control diet group, the abundances of *Methanimicrococcus* spp. in all fractions of the rhubarb treatment were significantly (*P* < 0.05) decreased on 50 d. In general, the addition of rhubarb numerically raised the abundances of Candidatus *Methanomethylophilus* spp. and reduced the abundances of *Methanomicrobium* spp. in all the fractions compared to the control on 50 d and 60 d.

**Table 3:** Comparison of relative abundance (%) of active methanogenic genera between control diet treatment and rhubarb supplementation treatment^a,b^.

Fraction	Diet	Age (d)		SEM^1^	Significance (P<)^2^		
		50	60		Diet	Age	Diet × Age
*Methanobrevibacter*							
RS	C^3^	36.89	36.95	0.149	<0.05	NS	NS
	R^4^	44.06	42.88				
RL	C	31.40^b^	13.93			<0.01	
	R	53.07^a^	15.64				
RP	C	40.17	35.67			NS	
	R	56.22	34.87				
RE	C	32.48	31.52			NS	
	R	39.71	27.39				
Candidatus *Methanomethylophilus*							
RS	C	13.43	28.57	0.376	NS	NS	NS
	R	31.23	31.94				
RL	C	14.77	53.15			<0.05	
	R	33.06	55.15				
RP	C	4.62	47.03			<0.01	
	R	22.53	39.52				
RE	C	23.45	16.98			NS	
	R	36.70	29.84				
*Methanosphaera*							
RS	C	15.23	16.26	0.130	NS	NS	NS
	R	16.04	7.79				
RL	C	5.10	3.22			NS	
	R	3.56	2.57				
RP	C	12.05	6.84			NS	
	R	13.49	10.18				
RE	C	20.32	35.39^a^			NS	
	R	18.06	23.79^b^				
*Methanomicrobium*							
RS	C	13.17	14.75	0.581	NS	NS	NS
	R	4.66	12.41				
RL	C	17.13	27.55			NS	
	R	6.43	23.45				
RP	C	1.83	2.82			NS	
	R	0.69	0.94				
RE	C	6.46	14.35			NS	
	R	1.95	12.92				
*Methanobacterium*							
RS	C	0.02	0.03	0.014	NS	NS	NS
	R	0.01	0.02				
RL	C	0.01	0.01			NS	
	R	0.01	0.01				
RP	C	0.01	0.01			NS	
	R	0.02	0.02				
RE	C	0.02	0.02			NS	
	R	0.01	0.02				
*Methanimcrococcus*							
RS	C	20.22^a^	2.57	0.151	<0.01	NS	<0.01
	R	2.53^b^	3.97				
RL	C	29.65^a^	1.24			<0.05	
	R	1.75^b^	2.59				
RP	C	40.09^a^	6.96			NS	
	R	5.39^b^	13.71				
RE	C	15.20^a^	0.85			NS	
	R	1.41^b^	4.99				

Means within a column for fractions that do not have a common superscript differ (P < 0.05). SEM ^1^represents SEM for fraction × age; ^2^NS = not significant (P > 0.05); ^3^C = control diet treatment; ^4^R = rhubarb supplementation treatment.

## Discussion

The initial colonization of methanogens in the rumen has been investigated using different methods in a few previous studies. Skillman *et al*.^35^ reported that the *Methanobrevibacter* spp. were detected in the rumen liquid of lambs at the age of 3 days by using PCR amplification. Using real-time qPCR, the existence of metabolically active methanogens (*Methanomicrobiales mobile*, *Methanoccocales votae*, and *Methanobrevibacter* spp.) was observed in the rumen of calves 20 minutes after birth^8^, and the presence of methanogenic archaea was found in the rumen of goats at 0 d^9^. More recently, Wang *et al*.^30^ used 16S rRNA sequencing to identify the colonization of methanogenic archaea in the rumen liquid of 7 day old goats. This is the first study, to our knowledge, that uses RNA based samples to investigate the development of the potentially active methanogens in the rumen. In the present research, results of real-time qPCR revealed that metabolically active methanogens initially colonized rumen both in RL and RE on the first day after birth, and this is supported by the findings of Jiao *et al*.^9^. It has been inferred that the maternal vagina and skin, the dam’s milk, or the surrounding environment could account for the initial colonization of microbes in the rumen^7, 36^. Methanogens utilize hydrogen as the source of energy to reduce carbon dioxide or acetate to methane during methanogenesis, and hydrogen is produced during carbohydrate fermentation in the rumen^17, 19^. It is assumed that proteobacteria (*Geobacter* spp.), *Ruminococcus flavefaciens*, or other species might provide methanogens with the essential hydrogen and electrons for methanogenesis during the early stage of rumen development before the ingestion of forage^8, 37^. In the current study, the active methanogenic archaeal populations in all the fractions began to increase and stabilize after the intake of starter concentrate. Starchy components in starter concentration promote H_2_ production, which helps for the methanogens colonization in the early stage of rumen development. It is noticeable that weaning did not affect the methanogen populations in all the four fractions, since no significant difference was observed between 38 d and 41 d. In addition, as the rumen gradually developed towards maturity and entered the phase of rumination (from 8 weeks onwards)^38, 39^, there were no differences of methanogen copy numbers in all fractions between 50 d and 60 d. Furthermore, the methanogen copy numbers in RS were continuously the highest among the four fractions, which is consistent with the previous findings^40, 41^ that the majority of the rumen microbiota is presented by those microbial populations associated to feed particles in rumen digesta, i.e., the solid-phase. Unlike the microorganisms in RS and RL, the microbes attached to the RE are not well characterized. In this study, the number of active methanogens in RE was the least compared to the other three fractions. A similar result has been reported by Liu *et al*.^29^. This result could be explained by the fact that RE is at the interface of host tissues and hence has less interaction with different feed, diverse microbes, and complex microscale activities than the microbes in the other fractions. In other ecosystems, the microbial density, diversity, and composition have been shown to be influenced by environmental heterogeneity^42^.

In this study, the age-dependent tendency for an increase in Chao1 richness was noted only in RE but not in the other three fractions that contained more methanogens. By contrast, it was reported that the Chao1 index of the bacterial community increased with age from 7 days to 2 years, while this pattern did not show in the archaeal community of the goats rumen^30^. The limited time span in this study (60 days) may be the reason why an age-dependent increment of Alpha diversity was not found in the RS, RL and RP fractions. Comparisons within four fractions at different days showed that the richness of the methanogen community in RE was lower than the other fractions for most of the time, which is in accordance with the methanogen densities found in this study. Further, no differences in Chao1 were found amongst the RS, RL, and RP, being supported by the finding of Belanche *et al*.^43^ that protozoa-associated methanogens and free-living methanogens share similar diversity indexes, since the rumen protozoa are continuously re-infected by the free-living methanogens within the rumen contents^44^. This does not preclude the possibility that individual species of protozoa may harbour specific methanogen populations^23^. PCoA of the methanogen community structure showed that in each fraction there was a remarkable gap between the communities before and post weaning, or between 38 d and 41 d, implying that weaning on 40 d had a significant impact on the structure of the rumen methanogenic archaeal community. Apart from the change of diet structure and components, weaning also involves both psychological and physiological stress as the kids were no longer raised together with the dams^45, 46^. However, as a strategy to adapt the suckling ruminants to a diet composed of forage and concentrates and reduce the cost of production, early weaning has been studied intensively and regarded as an effective approach in manipulating the microbial community and improving rumen fermentation^9, 47^. It was also revealed in the PCoA analysis that the methanogen community in RE was distinct from those in the other fractions for most of the time throughout rumen development, as supported by previous studies which reported differences between the epithelial tissue-associated and the rumen contents-associated bacterial communities^29, 48^.

The majority of previous research that has targeted the structure of the methanogen community reported that *Methanobrevibacter* phylotypes were the predominant methanogens in the rumen of different ruminants worldwide^28, 49–51^. In contrast a few studies found that the in the rumen of sheep, beef cattle, and reindeer, the archaeal community was dominated by the methanogenic archaea variably referred as “rumen cluster C (RCC)”, “uncultured novel order”, “Thermoplasmatales-Affiliated Lineage C (TALC)” or “order Methanoplasmatales”^52, 53^. Recently, those prevalent methanogenic archaea have been classified as the members from the seventh order of Thermoplasmatales, i.e., Methanomassiliicoccales^54, 55^. Compared to the DNA-based 16S rRNA gene sequencing, the RNA-derived amplicon sequencing could indicate the metabolically active microbes and their potential activities in rumen fermentation^31, 34^. In the present study, by the aid of the RNA-based 16S rRNA sequencing, we found that the *Methanobrevibacter* spp. and the genus Candidatus *Methanomethylophilus* which falls into the order Methanomassiliicoccales were generally the two most abundant active methanogens in the four fractions, followed by the *Methanosphaera* spp. and *Methanomicrobium* spp. with relatively lower abundances, while the members of the genera *Methanimicrococcus* and *Methanobacterium* successively occupied further minor proportions of the methanogen community. This finding is in line with conclusions in previous reports^43, 53^. Specifically, it was observed that the relative abundance of *Methanobrevibacter* spp. was the greatest in RP in contrast to the other fractions, which further verified that *Methanobrevibacter*-related methanogens dominate the methanogen populations attached to protozoa^24, 56^. In accordance with the conclusion of Tymensen *et al*.^24^ that the proportions of the *Methanomicrobium* spp. and RCC-related methanogens in the rumen protozoa fraction were lower than the free-living methanogens, it was shown in the present study that the minimum abundances of the genera *Methanomicrobium* and Candidatus *Methanomethylophilus* were both observed in RP compared with the other three fractions. In comparison with the rumen contents, there is more oxygen and urea in the ruminal epithelium as the juncture of ruminant tissue and rumen digesta^57^. This study revealed that the fraction of RE harbored more *Methanosphaera*-related methanogens than the other three fractions, and it could be hypothesized that *Methanosphaera* spp. is more capable of enduring the oxygen toxicity and colonizing in this specific environment, but the mechanism is unclear and hence requires further investigations. The relative abundances of all the methanogenic genera except *Methanobacterium*, which accounted for an insignificant proportion, experienced sequential dramatic fluctuations, implying that the methanogen community was unstable and changeable in response to the accumulation of age, the shift in diet, and the stress of weaning during rumen development. This could be a result of competition between different genera for hydrogen or other substrates of methanogenesis^58, 59^. During rumen development, each methanogenic genus generally changed in the same manner despite the discrepancies of relative abundances across fractions, which indicated that age, diet, and weaning impose significant influences on the methanogen community in all of the fractions.

Effects of rhubarb treatment on rumen fermentation were examined in a few studies *in vitro* and *in vivo*, which suggested that rhubarb could regulate rumen fermentation by reducing methane production and the acetate: propionate ratio^60, 61^. In the present study, rhubarb was supplemented to the starter diet of black goats along with early weaning, and it was observed that although the densities of the potentially active methanogens were not affected, the addition of rhubarb raised the abundance of *Methanobrevibacter* spp. in the fraction of RE but more significantly decreased the abundance of *Methanimicrococcus* phylotypes in all the four fractions. In contrast, Kim *et al*.^26^ found no significant difference in the relative abundance of *Methanobrevibacter* between the samples before and after the inclusion of rhubarb in the rumen of steers. This inconsistency might be ascribed to the differences between the species of rhubarb and/or the hosts used in the two studies and needs further investigations to be explained. Since references on the effect of rhubarb on methanogenic community are limited, it is assumed that the incremental abundance of *Methanobrevibacter* spp. in this research could be explained by the dramatic decline of *Methanimicrococcus*-related methanogens, as the former genus is regarded as the most prevalent methanogenic genus in rumen. Rhubarb contain snthraquinone derivatives of rhein, emodin, and aloe-emodin, and may directly suppress methanogenic archaea and the utilization of hydrogen, leading to the greatly increased headspace hydrogen gas accumulation^62, 63^. Further, almost no significant differences of the relative abundances of methanogenic genera between rhubarb treatment and the control group were noted on 60 d in this study, indicating the potential for rumen microbial adaptation and/or the room for improving rhubarb dosage^60, 61, 64^.

In the present study, individual variation was noted in initial establishment as well as the subsequent evolution of the methanogens across four fractions. The sources of the initial colonization of rumen microbiota and their actual effects might differ amongst the individuals, and the genetic influence of the host could result in the individual variations in the microbial establishment and development^7, 36, 65^. Findings of previous studies on the comparison of methanogen communities between ruminants with different methane emissions are somewhat inconsistent. Shi *et al*.^66^ described no difference in archaeal metagenomic abundances, but differences in metatranscriptomic abundances between high and low methane emitting sheep. By contrast, another group reported disparities in archaeal metagenomic abundances between high and low methane yielding cattle^67, 68^. Furthermore, Kittelmann *et al*.^69^ observed no differences in archaeal community but did note some differences in the relative abundances of the bacterial genera *Quinella* and *Sharpea* between high- and low-CH_4_ emitting sheep. In this research, the alteration by rhubarb on the composition of methanogen community was present, suggesting more research should be performed to further explore its effect on the methanogenesis pathways and the detailed mode of action.

In the current study, the initial colonization of metabolically active methanogens was observed by qPCR in RL and RE on the first day after birth, and afterwards the methanogenic densities in four fractions gradually became stable as solid feed was introduced and the goats were early weaned. However, the variability and instability of the potentially active methanogen community composition corresponding to the change of diet and age was also observed. In addition, the diversities and structures of methanogenic populations, and the distributions of methanogenic genera differed within the four tractions during the development of the rumen, implying that the disparity across these four fractions should be taken into consideration when investigating the overall methanogen community and methanogenesis. This study contributes to the knowledge of the development of the rumen methanogen community and relevant modulation, and mitigation of methane production during rumen development. Future investigations should aim at the interactions of the anatomical, functional, and microbial development, as well as the impact of manipulation during early life on ruminant production in the long term.

## Methods

All procedures for animal experiment were conducted according to the guidelines approved by the Animal Care Committee (Approval Number: 20140206), Institute of Subtropical Agriculture, Chinese Academy of Sciences, Changsha, China. The principles of laboratory animal care were met and slaughter procedures were performed in accordance with the guidelines of Chinese national standards of cattle and goat slaughtering by reducing the animal suffering as much as possible. All experimental protocols were also approved by Institute of Subtropical Agriculture, Chinese Academy of Sciences, Changsha, China.

### Animals, diets and management

Forty-five newborn Xiangdong black goats (*Capra hircus*) used in this study were housed in a well-ventilated room with controlled temperature and humidity. The experimental start for each goat was staggered to accommodate differing birth dates. After birth, the goats were left with their dams until weaning. On 1, 10, and 20 d, 8, 7, and 6 goats were slaughtered respectively. The remaining goats were gradually weaned off goat milk and supplied with free access to a mixture of fresh grass (*Miscanthus sinensis*, 40% of total dry matter [DM]) and starter concentrate (60% of total DM) from 15 d until they were weaned at 40 d. Four goats were further slaughtered at 38 d and 41 d, respectively. Sixteen goats were randomly assigned to two diet treatments: the control diet and the diet supplemented with rhubarb (*Rheum offcinale* Baill.) root powder, and then reared separately from the dams after weaning.

The control diet (per kg DM) contained 400 g fresh grass (in DM) and 600 g starter concentrate (in DM), and every 600 g starter concentrate was composed of the following components: 193 g extruded soybean, 69 g whey powder, 100 g maize flour, 109 g fat powder, 80 g soybean meal, 6 g CaCO_3_, 15 g CaHPO_4_, 8 g NaCl, and 20 g premix. In the control treatment, goats were fed 150 g control diet twice per day at 08.00 and 17.00 h, and four goats were slaughtered separately at 50 d and 60 d. In the rhubarb supplemented group, goats were gradually accustomed to the supplementation of rhubarb from one week before weaning. Two goats were removed for the reason irrelevant to the experiment, the remaining six goats received 150 g control diet plus 2 g rhubarb root powder per meal, and three goats were slaughtered at 50 d and 60 d, respectively. The management of goats and sampling is further illustrated in Supplementary Fig. S5, and the increase of body weight is shown in Supplementary Fig. S6.

### Sample fractionation

After the goats were slaughtered, the rumen was immediately removed for sampling of the four fractions, i.e., the RS, RL, RP, and RE. RS and RL samples were collected and separated using a French press filter (Bodum Inc., Triengen, Switzerland) according to the method described by Kong *et al*.^70^. To obtain the RP samples, 10 mL of rumen fluid was centrifuged at 500 g for 1 min and the protozoal pellet was then rinsed with sterile anaerobic saline solution and collected by centrifugation (500 × g) for 3 times. Three RP samples from each goat were pooled for analysis. For the RE samples, 3 pieces of 2 g (approximately 4 cm^2^) epithelium samples were excised at different sites of the same rumen and washed with sterile saline solution and then combined. As the rumen was underdeveloped and the contents were limited, no RS and RP sample was collected on 1 and 10 d, and only 4 RL samples were collected on 1 d. Five RS samples and 4 RP samples were collected on 20 d. All the samples were immediately flash-frozen in liquid nitrogen and then stored at −80 °C for subsequent use.

### RNA extraction and first-strand cDNA synthesis

To isolate total RNA a modification of the method described by Wang *et al*.^22^ was used. Briefly, samples were first manually ground into crude powder in liquid nitrogen using a mortar and pestle, and then 2 g of crude powder was respectively weighed and further ground for 5 min in liquid nitrogen using a Retsch RM100 grinder (Retsch GmbH, Haan, Germany). After grinding, 0.3 g frozen fine powder was weighed into each 50-mL tube and mixed with 3 mL of Ambion TRIzol reagent (Life Technologies, Carlsbad, USA). Subsequent procedures were conducted in accordance with the method of Wang *et al*.^22^. After the extraction, an Ambion MEGAclear kit (Life Technologies, Carlsbad, USA) was used to purify the isolated RNA. The RNA concentration and integrity were estimated using an Agilent 2100 bioanalyzer and RNA 6000 Nano kit (Agilent Technologies, Santa Clara, USA). The prokaryotic total RNA nano assay protocol was used, as prokaryotes account for the majority of RNA in rumen contents^40^.

Five hundred ng of isolated total RNA from each sample was used to synthesize the first-strand cDNA using an Invitrogen SuperScript III RT kit (Life Technologies, Carlsbad, USA), and the cDNA synthesis reactions were stored at −20 °C until further analysis was performed.

### Real-time quantitative PCR

To estimate the methanogen 16S rRNA copy number of each sample, qPCR was conducted as described by Ohene-Adjei *et al*.^71^ and Hristov *et al*.^72^ with modifications. The qPCR was performed on a 96-well ABI 7900HT (Applied Biosystems, Foster City, USA), and the archaeal specific primers Arch 1174–1195 F (5′-GAGGAAGGAGTGGACGACGGTA-3′) and Arch 1406–1389 R (5′-ACGGGCGGTGTGTGCAAG-3′) were used for the amplification of the serially diluted standards and the cDNA samples^71^. Each reaction mix (10 μL) consisted of 1 μL standard DNA or diluted first-strand cDNA, 1 μL of each primer (5 μM), 2 μL molecular biology grade H_2_O, and 5 μL DyNAmo HS SYBR Green qPCR 2 × master mix (Thermo Fisher Scientific, Waltham, USA). The qPCR cycling conditions were 40 cycles of 95 °C for 15 s, and 60 °C for 60 s. The linear relationship observed between the threshold amplification (*C*_t_) and the logarithm of 16S rRNA copy numbers of the standards was used to calculate the copy numbers of methanogens per μL of cDNA. Each estimate was a mean of triplicates.

### PCR amplification and 16S rRNA amplicon sequencing

The PCR amplification of archaeal 16S rRNA genes was conducted on a Dyad Peltier Thermal Cycler (AL056543, Bio-Rad Laboratories, Hercules, USA) using specific primers Ar915aF (5′-AGGAATTGGCGGGGGAGCAC-3′) and Ar1386R (5′-GCGGTGTGTGCAAGGAGC-3′) described by Kittleman *et al*.^73^ with modifications. A dual barcode assay adapted for the Illumina MiSeq sequencer (Illumina Inc., San Diego, USA) was used (see Supplementary Table S2). Each primer contained the Illumina adapter sequence, unique barcode, spacer and forward or reverse primer. For each cDNA sample, 20 μL of reaction mix was prepared containing 1 μL cDNA, 1 μL of each barcoded primer (1 μM), 7 μL molecular biology grade H_2_O, and 10 μL KAPA2G Robust Hotstart ReadyMix (Kapa Biosystems, Wilmington, USA). The PCR procedures were as follows: initial denaturation at 95 °C for 5 min; 30 cycles of denaturation (95 °C, 20 s), annealing (55 °C, 15 s) and elongation (72 °C, 5 min); and a final 10-min extension at 72 °C. Each cDNA sample was amplified in duplicates, and 3 wells per run served as a negative control for the master mix. After amplification, duplicate PCR products were pooled, and the correct sizes of PCR products and the absence of signal from negative controls were further verified through agarose gel electrophoresis. Quantitation of amplicons was performed in a Synergy HTX Multi-Mode Microplate Reader (model SIAFRM, Bio-Tek Instruments Inc., Winooski, USA) using a Quant-iT dsDNA Assay Kit (Thermo Fisher Scientific, Waltham, USA). The amplicons were pooled in equimolar concentrations and purified using Agencourt AMPure XP beads (Beckman Coulter Inc., Brea, USA) and then further quantified as described above. The amplicon library was combined with 5% PhiX control library and sequenced in the Illumina Miseq (Illumina Inc., San Diego, USA).

### Bioinformatic analysis

The quality of the raw fastq files were checked with the FastQC program (http://www.bioinformatics.bbsrc.ac.uk/projects/fastqc). Trimmomatic v0.33^74^ was used to trim the raw reads, to remove ambiguous and low quality reads. Reads with average quality score <20 over a 4 bp sliding window and reads with lengths shorter than 36 bp were removed. Merging of the paired-end reads was effected with PEAR v0.9.8 using default options^75^. Reads which did not get assembled were discarded. High quality sequence reads from the various samples were then combined into a single dataset and subsequent analysis was carried out using the open-source software package, QIIME V1.8.0^76^. This primarily involved picking Operational Taxonomic Units (OTUs), assigning taxonomy, inferring phylogeny, creating OTU tables and computing microbial community diversity indices. The sequences were clustered into Operational Taxonomic Units (OTUs) using the *de novo* OTU picking workflow with a 97% similarity threshold. Taxonomic assignment of OTUs was performed by comparing the most abundant ‘representative sequences’ within each OTU to the SILVA v119 database^77^. To enable calculation of Unifrac distances^78^ and to facilitate downstream diversity analysis the picked OTUs were aligned by PyNAST^79^ against the core alignment template of SILVA v119, and a phylogenetic tree was built using FastTree^80^. To differentiate the conserved from the non-conserved regions of the alignment and remove sections comprised of only gaps (useful in phylogenetic tree construction) a lanemask file was applied. This was constructed from the SILVA v119 core alignment file using a python script. The alpha (within sample) diversity of the samples was estimated using the Chao1, Shannon and observed_otus indices. The Chao1 index was used to further compare the alpha diversity of the samples. Beta (between sample) diversity of the samples was also computed and visualized with three dimensional principal coordinate analysis (PCoA) plots generated using the Bray-Curtis dissimilarity index^81^ and the unweighted UniFrac distances. Information on the summary of sequencing data is displayed in the Supplementary Table S3. All the sequences in the present study were deposited to the sequence read archive (SRA) of the NCBI database using files generated by Mothur V1.33.3^82^, under the accession number SRP080922.

### Statistical analysis

Data obtained from qPCR were analyzed as a completely randomized design using the PROC MIXED procedure of SAS (SAS Institute, 2001) to test the effect of fractions of samples, the model included fraction, age, and fraction × age as the fixed effects, with individual animal as the experimental unit. To test the effect of age on the copy number of methanogens, the PROC MIXED procedure of SAS (SAS Institute, 2001) was used, with animal nested within age as the random effect and individual animal as the experimental unit. Linear, quadratic, and cubic effects of age were analyzed using orthogonal polynomial contrasts. To compare the copy numbers of methanogens between the control diet group and the rhubarb group, the PROC MIXED procedure of SAS (SAS Institute, 2001) was used with a model which included the fixed effects of diet, age and diet × age interaction, with individual animal as the experimental unit. For the analysis of relative abundance data at genus level, the compliance of data with the assumptions of normality and homogeneity of variances was first examined visually through residual plots created by the UNIVARIATE and PLOT procedures (SAS Institute, 2001), and variables that were deemed non-normal were then arcsine transformed. To test the effects of sample fractions, age, and the addition of rhubarb, the PROC MIXED procedure of SAS (SAS Institute, 2001) was used separately in the methods as described above. Least squares means are reported throughout the text, and statistical significance was declared at P < 0.05.

## Electronic supplementary material

Supplementary Information
