# Supplementary material for: Investigation and manipulation of metabolically active methanogen community composition during rumen development in black goats
Source: Sci Rep. 2017 Mar 24;7:422. doi: 10.1038/s41598-017-00500-5 (PMC5428682; doi:10.1038/s41598-017-00500-5)

Supplementary Information

Investigation and manipulation of metabolically active methanogen community composition during rumen development in black goats

Zuo Wang [1,2,3], Chijioke Elekwachi [3], Jinzhen Jiao [1], Min Wang [1*], Shaoxun Tang [1], Chuanshe Zhou [1], Zhiliang Tan [1], and Robert J. Forster [3*]

[1] Key Laboratory for Agro-Ecological Processes in Subtropical Region, Hunan Research Center of Livestock & Poultry Sciences, South-Central Experimental Station of Animal Nutrition and Feed Science in Ministry of Agriculture, Institute of Subtropical Agriculture, Chinese Academy of Sciences, Changsha, Hunan 410125, China

[2] University of Chinese Academy of Sciences, Beijing 100049, China

[3] Lethbridge Research and Development Centre, Agriculture and Agri-Food Canada, Lethbridge, AB T1J 4B1, Canada

* Corresponding authors: Min Wang. Address: Institute of Subtropical Agriculture, the Chinese Academy of Sciences, Changsha, Hunan 410125, China. E-mail: mwang@isa.ac.cn; Tel: (86)-731-84619702; Fax: (86)-731-84612685. Robert J. Forster. Address: Lethbridge Research and Development Centre, Agriculture and Agri-Food Canada, Lethbridge, AB T1J 4B1, Canada. E-mail:robert.forster@agr.gc.ca; Tel: (001)-403-317-2292; Fax: (001)-403-382-3156.

Contents:

Supplementary Tables

Supplementary Figures

Supplementary Table S1. Comparison of methanogen copy numbers (Log_{10} copies/ µL cDNA) between control diet treatment and rhubarb supplementation treatment SEM [1] represents SEM for fraction × age; [2] NS = not significant (P > 0.05); [3] C = control diet treatment; [4] R = rhubarb supplementation treatment.

Fraction	Diet	Age (d)		SEM[1]	Significance (P <)[2]		
		50	60		Diet	Age	Age × Diet
RS	C[3]	8.9890	8.4584	0.2173	NS	NS	NS
	R[4]	8.6309	8.9187				
RL	C	6.9032	6.0128			NS	
	R	6.2425	5.5796				
RP	C	7.4837	7.4950			NS	
	R	7.9907	7.4648				
RE	C	6.1004	5.8813			NS	
	R	5.4840	5.4740				

Supplementary Table S2. Dual index amplicon primers for methanogens.

Code	Sequence	Barcode
MARead1	TATGGTAATT GG AGGAATTGGCGGGGGAGCAC	
MA.Read2	AGTCAGTCAGCC GCGGTGTGTGCAAGGAGC	
MA.Index	GCTCCTTGCACACACCGCGGCTGACTGACT	
MA.SA501	AATGATACGGCGACCACCGAGATCTACACATCGTACGTATGGTAATTGGAGGAATTGGCGGGGGAGCAC	SA501 ATCGTACG
MA.SA502	AATGATACGGCGACCACCGAGATCTACACACTATCTGTATGGTAATTGGAGGAATTGGCGGGGGAGCAC	SA502 ACTATCTG
MA.SA503	AATGATACGGCGACCACCGAGATCTACACTAGCGAGTTATGGTAATTGGAGGAATTGGCGGGGGAGCAC	SA503 TAGCGAGT
MA.SA504	AATGATACGGCGACCACCGAGATCTACACCTGCGTGTTATGGTAATTGGAGGAATTGGCGGGGGAGCAC	SA504 CTGCGTGT
MA.SA505	AATGATACGGCGACCACCGAGATCTACACTCATCGAGTATGGTAATTGGAGGAATTGGCGGGGGAGCAC	SA505 TCATCGAG
MA.SA506	AATGATACGGCGACCACCGAGATCTACACCGTGAGTGTATGGTAATTGGAGGAATTGGCGGGGGAGCAC	SA506 CGTGAGTG
MA.SA507	AATGATACGGCGACCACCGAGATCTACACGGATATCTTATGGTAATTGGAGGAATTGGCGGGGGAGCAC	SA507 GGATATCT
MA.SA508	AATGATACGGCGACCACCGAGATCTACACGACACCGTTATGGTAATTGGAGGAATTGGCGGGGGAGCAC	SA508 GACACCGT
v4.SA701	CAAGCAGAAGACGGCATACGAGATAACTCTCGAGTCAGTCAGCCGCGGTGTGTGCAAGGAGC	SA701 AACTCTCG
v4.SA702	CAAGCAGAAGACGGCATACGAGATACTATGTCAGTCAGTCAGCCGCGGTGTGTGCAAGGAGC	SA702 ACTATGTC
v4.SA703	CAAGCAGAAGACGGCATACGAGATAGTAGCGTAGTCAGTCAGCCGCGGTGTGTGCAAGGAGC	SA703 AGTAGCGT
v4.SA704	CAAGCAGAAGACGGCATACGAGATCAGTGAGTAGTCAGTCAGCCGCGGTGTGTGCAAGGAGC	SA704 CAGTGAGT
v4.SA705	CAAGCAGAAGACGGCATACGAGATCGTACTCAAGTCAGTCAGCCGCGGTGTGTGCAAGGAGC	SA705 CGTACTCA
v4.SA706	CAAGCAGAAGACGGCATACGAGATCTACGCAGAGTCAGTCAGCCGCGGTGTGTGCAAGGAGC	SA706 CTACGCAG
v4.SA707	CAAGCAGAAGACGGCATACGAGATGGAGACTAAGTCAGTCAGCCGCGGTGTGTGCAAGGAGC	SA707 GGAGACTA
v4.SA708	CAAGCAGAAGACGGCATACGAGATGTCGCTCGAGTCAGTCAGCCGCGGTGTGTGCAAGGAGC	SA708 GTCGCTCG
v4.SA709	CAAGCAGAAGACGGCATACGAGATGTCGTAGTAGTCAGTCAGCCGCGGTGTGTGCAAGGAGC	SA709 GTCGTAGT
v4.SA710	CAAGCAGAAGACGGCATACGAGATTAGCAGACAGTCAGTCAGCCGCGGTGTGTGCAAGGAGC	SA710 TAGCAGAC
v4.SA711	CAAGCAGAAGACGGCATACGAGATTCATAGACAGTCAGTCAGCCGCGGTGTGTGCAAGGAGC	SA711 TCATAGAC
v4.SA712	CAAGCAGAAGACGGCATACGAGATTCGCTATAAGTCAGTCAGCCGCGGTGTGTGCAAGGAGC	SA712 TCGCTATA
MA.SB501	AATGATACGGCGACCACCGAGATCTACACCTACTATATATGGTAATTGGAGGAATTGGCGGGGGAGCAC	SB501 CTACTATA
MA.SB502	AATGATACGGCGACCACCGAGATCTACACCGTTACTATATGGTAATTGGAGGAATTGGCGGGGGAGCAC	SB502 CGTTACTA
MA.SB503	AATGATACGGCGACCACCGAGATCTACACAGAGTCACTATGGTAATTGGAGGAATTGGCGGGGGAGCAC	SB503 AGAGTCAC
MA.SB504	AATGATACGGCGACCACCGAGATCTACACTACGAGACTATGGTAATTGGAGGAATTGGCGGGGGAGCAC	SB504 TACGAGAC
MA.SB505	AATGATACGGCGACCACCGAGATCTACACACGTCTCGTATGGTAATTGGAGGAATTGGCGGGGGAGCAC	SB505 ACGTCTCG
MA.SB506	AATGATACGGCGACCACCGAGATCTACACTCGACGAGTATGGTAATTGGAGGAATTGGCGGGGGAGCAC	SB506 TCGACGAG
MA.SB507	AATGATACGGCGACCACCGAGATCTACACGATCGTGTTATGGTAATTGGAGGAATTGGCGGGGGAGCAC	SB507 GATCGTGT
MA.SB508	AATGATACGGCGACCACCGAGATCTACACGTCAGATATATGGTAATTGGAGGAATTGGCGGGGGAGCAC	SB508 GTCAGATA

Supplementary Table S3. Summary of sequencing data.

Note: All sequenced samples had a RIN value⩾8.							
Sample	Archaeal sequences	Observed_OTUs	Good's coverage	PD_whole_tree	Shannon	Simpson	Chao1
RS20.1	19968	5124	0.777994792	97.67472	6.6300134	0.868649078	30442.37113
RS20.2	19620	4235	0.814576962	92.11002	5.91455497	0.861045439	22766.38095
RS20.3	18615	3703	0.832661832	81.75395	6.15344087	0.906350816	18906.93417
RS20.5	19698	4750	0.796273733	102.72981	6.90085274	0.919625275	26389.99462
RL20.6	19662	4370	0.813447259	89.6991	6.42011815	0.888324137	24208.57817
RS38.1	19930	3974	0.827094832	88.36312	5.22954033	0.784966605	22998.79167
RS38.2	19869	3816	0.835522674	84.77352	5.08225021	0.788157808	19846.86486
RS38.3	19920	4493	0.810040161	97.77648	6.90902777	0.92479527	24942.81714
RS38.4	19927	3865	0.835750489	87.86181	4.88322104	0.741656618	18656.79006
RS41.1	19900	3925	0.83120603	92.81598	5.58726942	0.839454923	21385.56037
RS41.3	19913	3765	0.834279114	92.72172	5.84345387	0.901587224	23925.55556
RS41.4	19928	4296	0.812826174	102.23677	6.41455697	0.924779608	24454.21739
RS50.1	19822	3661	0.840076682	89.23386	5.66460116	0.896695859	20348.25914
RS50.2	19955	3629	0.840240541	89.55929	5.54069873	0.88855184	24364.01224
RS50.3	19926	3638	0.841112115	87.37	5.14810723	0.80482683	21341.86926
RS50.4	19691	3681	0.837438424	88.6981	5.66936591	0.894374988	21651.52632
RS60.1	19971	4240	0.816383756	97.78417	6.2444095	0.897885966	27742.13636
RS60.2	19763	3747	0.836917472	88.50321	5.49492141	0.848461095	22906.60517
RS60.3	19868	3771	0.838634991	90.77904	5.70322742	0.876699251	19676.92879
RS60.4	19755	4104	0.824702607	96.35611	6.32777009	0.90575717	20801.64067
RS50R.1	19919	4159	0.8177	99.73763	5.77132757	0.877544295	25456.54808
RS50R.2	19836	4262	0.8131	102.91814	5.84498076	0.86214894	26792.49355
RS50R.3	19929	4033	0.8238	99.9458	5.48650769	0.83920495	24385.54426
RS60R.1	19597	4859	0.7935	118.80962	6.32374845	0.840792545	27179.37958
RS60R.2	19950	4445	0.8058	109.39789	5.7001764	0.798001565	28083.82759
RS60R.3	19908	3798	0.83465	96.61388	5.51805237	0.858100485	23251.63345
RL1.4	17819	4931	0.761995623	103.9779	7.0147111	0.887499296	29165.28571
RL1.6	14758	4359	0.746239328	90.78841	6.97468885	0.864394459	24562.57349
RL1.7	4119	1244	0.724447681	30.01824	5.97411188	0.882211833	10849.14925
RL1.8	1378	468	0.692307692	12.58724	5.55058551	0.876416885	4366.956522
RL10.1	19308	4830	0.786824114	100.44196	6.20450319	0.823159563	26999.29319
RL10.2	17531	4458	0.78557983	93.72509	6.20275122	0.793870484	23756.2541
RL10.3	18195	3508	0.836328662	82.33595	4.20533408	0.549595419	17715.54167
RL10.4	19818	5186	0.775456656	108.63497	6.79436525	0.899032762	28047.48961
RL10.5	19523	4902	0.783281258	108.22449	6.17503866	0.793647769	29828.3649
RL10.6	18107	5404	0.745954603	114.53023	6.8029011	0.801460208	30709.50239
RL10.7	19568	4934	0.781888798	104.0094	6.30623741	0.845078162	31327.55942
RL20.1	18749	4664	0.789802123	100.54643	6.17836326	0.803232308	23928.93797
RL20.2	19494	5020	0.776290141	112.94309	6.61064892	0.882491159	32338.90805
RL20.3	19672	5355	0.766012607	115.63167	7.30847661	0.927127833	33009.05483
RL20.4	14450	4131	0.758200692	92.00463	7.60071708	0.931856582	24746.78041
RL20.5	19614	5305	0.778270623	114.3287	7.30629563	0.897057328	25002.34583

RL20.6	19665	4667	0.79913552	102.21741	6.55328416	0.892629329	28591.15644
RL38.1	19937	4877	0.791743994	103.10575	6.1538803	0.837199097	25058.44262
RL38.2	19510	4883	0.785699641	101.03213	6.21489112	0.845748506	25347.37939
RL38.3	18068	4723	0.779555014	98.8205	7.35159487	0.923146582	24698.19647
RL38.4	19230	4834	0.785335413	103.43476	6.11291613	0.807648313	24597.63805
RL41.1	19795	4327	0.811568578	99.16738	5.70296413	0.826899524	24543.81686
RL41.2	19855	4345	0.812087635	98.07186	6.09733775	0.876399269	24631.63265
RL41.3	19853	4369	0.81091019	103.04566	6.41109619	0.904732527	23204.24332
RL41.4	19859	4643	0.800140994	103.55122	6.38658298	0.862396102	25811
RL50.1	19041	3611	0.83703587	85.49753	5.5159767	0.854616319	20036.77816
RL50.2	19727	3377	0.849039388	82.08482	4.92324112	0.828881208	21108.012
RL50.3	18830	3718	0.82968667	87.03949	5.58824233	0.834752862	20518.06863
RL50.4	18524	4042	0.809868279	90.22609	5.99033607	0.891041292	24043.55161
RL60.1	17434	4276	0.787254789	94.29575	5.99215585	0.791209382	25565.43034
RL60.2	16172	3242	0.825500866	77.75912	5.40899989	0.801547315	18551.35
RL60.3	19819	4019	0.826176901	93.4682	5.21230135	0.759349834	21065.81034
RL60.4	17973	4248	0.801535637	93.45577	6.11446	0.7673905	22063.01681
RL50R.1	19879	5327	0.76685	108.82107	6.83019012	0.88327447	36924.24709
RL50R.2	19370	4564	0.80135	113.07722	5.77509175	0.821125545	27172.53295
RL50R.3	16338	3448	0.8544	85.22284	5.90999194	0.869396375	18216
RL60R.1	19224	5008	0.7874	122.04957	6.82558391	0.865015735	28421.53886
RL60R.2	19113	5017	0.7869	118.12161	6.69422773	0.859192195	27717.4775
RL60R.3	5888	1197	0.835368755	36.75085	4.64883717	0.722232082	7312.943182
RP20.2	19946	5280	0.779003309	111.96207	6.81385897	0.855896999	25473.40541
RP20.3	18998	4496	0.801242236	102.40631	6.95703019	0.929619549	24076.21978
RP20.5	19964	4794	0.798988179	107.53898	6.9329193	0.913551651	25277.65903
RP20.6	19878	4723	0.802092766	105.81183	6.69329204	0.892710809	23545.89781
RP38.1	19671	4150	0.820141325	96.11653	5.26347408	0.717973555	21725.71067
RP38.2	19830	4313	0.813968734	97.43785	5.37024525	0.752336713	23804.44986
RP38.3	19838	4671	0.802096986	104.4017	6.97163687	0.919824116	24376.30691
RP38.4	19562	3480	0.8538493	83.63571	4.49295417	0.599687512	14765.94199
RP41.1	19721	4432	0.811368592	98.9158	6.05740553	0.801406999	22078.27551
RP41.2	19857	3413	0.856675228	78.51057	4.51335411	0.634965389	15497.8806
RP41.3	19949	4432	0.808311194	104.92466	6.26850056	0.895960699	25930.75294
RP41.4	19947	4642	0.799318193	108.33121	6.50496944	0.906404956	25339.68217
RP50.1	19801	3568	0.847027928	86.78042	5.33362045	0.810249024	17943.88088
RP50.2	19952	3480	0.850791901	83.62952	5.08340295	0.816687747	18099.72277
RP50.3	19935	3743	0.838274392	89.26596	5.08817511	0.803238446	20611.42857
RP50.4	19257	4622	0.797735888	109.33845	6.46253164	0.873533663	22808.00719
RP60.1	18336	3960	0.814626963	97.60942	5.78196642	0.813807651	22351.40446
RP60.2	16447	3189	0.830729008	77.20689	5.49987557	0.824117667	19397.93724
RP60.3	19847	4283	0.818511614	102.61681	5.98404453	0.825684141	21084.55699
RP60.4	19489	4423	0.803324953	107.10819	6.39004335	0.8998143	27517.42767
RP50R.1	19928	4261	0.8157	101.03029	5.33232136	0.735302875	25093.68405
RP50R.2	19859	4574	0.79895	109.49342	5.96759647	0.86486709	29366.05521
RP50R.3	19866	4159	0.8181	101.77661	5.39136685	0.816320355	26661.39116

RP60R.1	19257	5464	0.7614	136.99837	6.99323744	0.91230531	33502.43842
RP60R.2	19840	4844	0.78795	116.38394	5.95387387	0.83763839	29143.78378
RP60R.3	15708	3602	0.8435	99.65176	6.1340372	0.91766687	20429.78351
RE1.1	753	276	0.665338645	8.06849	5.57359124	0.913967856	3438.6
RE1.2	645	226	0.691472868	6.42644	5.14338509	0.859232017	1867.75
RE1.3	781	272	0.693982074	7.38593	5.30975195	0.87301975	2049.5625
RE1.4	622	190	0.726688103	5.81662	4.7868059	0.862124048	2584.166667
RE1.5	203	74	0.669950739	2.82009	4.2511278	0.837778155	2285
RE1.6	385	140	0.677922078	4.28463	5.09973382	0.91255861	1411
RE1.7	578	114	0.822827939	4.75523	3.31891681	0.716413839	1185.2
RE1.8	78	40	0.538461538	1.88877	4.18155887	0.872781065	355
RE10.1	12293	2918	0.803465387	62.18466	5.50850992	0.738500705	13565.15328
RE10.2	685	216	0.709489051	5.76039	4.96201222	0.8918749	2405
RE10.3	5603	1454	0.768873818	35.09602	5.6263376	0.861297597	10763.61111
RE10.4	6251	1449	0.791393377	34.04721	4.82876847	0.781657613	10994.57303
RE10.5	1452	417	0.732093664	10.53838	4.24485635	0.717456306	5448.066667
RE10.6	839	294	0.675804529	7.50167	4.74166169	0.789241975	3129.076923
RE10.7	788	270	0.680203046	6.24898	4.34855692	0.716512278	3145.090909
RE20.1	8773	1843	0.818762111	43.5946	5.2347126	0.82187071	11063.83942
RE20.2	7981	2158	0.770956021	48.0313	5.99369889	0.81822533	11645.94318
RE20.3	4581	1223	0.76315215	30.28031	5.9790584	0.908972889	9390.638889
RE20.4	1405	433	0.732384342	10.91814	5.74181498	0.893847849	2783
RE20.5	2478	795	0.719935432	18.94292	6.14816626	0.919221677	4941.051724
RE20.6	3233	944	0.749149397	20.90895	5.76198509	0.865990407	5570.126761
RE38.1	18015	3519	0.830585623	81.47429	5.23140061	0.823998178	20087.7758
RE38.2	4953	1067	0.807389461	25.01325	4.90010234	0.84765738	7294.136986
RE38.3	15823	3510	0.811540163	80.17534	6.51776605	0.921820578	18525.78041
RE38.4	18407	4123	0.812245341	87.6725	5.9938213	0.860349592	20215.29111
RE41.1	16448	3092	0.837609436	73.65451	4.99215449	0.778271388	16153.48352
RE41.2	13051	2629	0.821699487	61.66031	5.26885149	0.843056006	17498.78571
RE41.3	13232	2833	0.80887243	70.6316	5.95813389	0.919632922	19836.48936
RE41.4	9209	1905	0.813009013	48.01209	5.572668	0.901173755	14253.175
RE50.1	17835	3452	0.830613961	82.04119	5.49472649	0.865690365	20731.20455
RE50.2	13677	2386	0.845726402	60.06225	5.13639258	0.886316093	15100.25714
RE50.3	16019	3244	0.831200449	76.18178	6.81032201	0.936091736	17861.824
RE50.4	16793	3727	0.808789377	84.49959	6.10688252	0.901224688	20792.08278
RE60.1	14572	3563	0.788086742	81.59319	6.67009987	0.91617026	21965.81081
RE60.2	18014	3424	0.837681803	81.07145	5.30923946	0.837625773	18366.04895
RE60.3	16082	3521	0.807711505	81.43344	5.81384904	0.872385285	21852.35521
RE60.4	18028	3259	0.845518083	78.27065	5.05118903	0.81359673	17204.03597
RE50R.1	11725	4140	0.8256	83.50806	6.85702732	0.94318104	21921.66082
RE50R.2	8076	2946	0.87545	65.3579	5.66119912	0.89541943	16371.51948
RE50R.3	17140	4229	0.8148	104.04129	6.00911109	0.881679435	26714.10164
RE60R.1	13074	4231	0.82195	100.48268	6.40639455	0.887999025	21838.16667
RE60R.2	15105	4483	0.81575	101.72639	6.51220464	0.86037414	20644.35714
RE60R.3	18055	4233	0.8169	105.59254	6.23331229	0.894760285	24065.22189

Supplementary Figure S1. Composition of methanogen communities at genus level in RS (A), RL (B), RP (C), and RE (D) across individuals during rumen development.

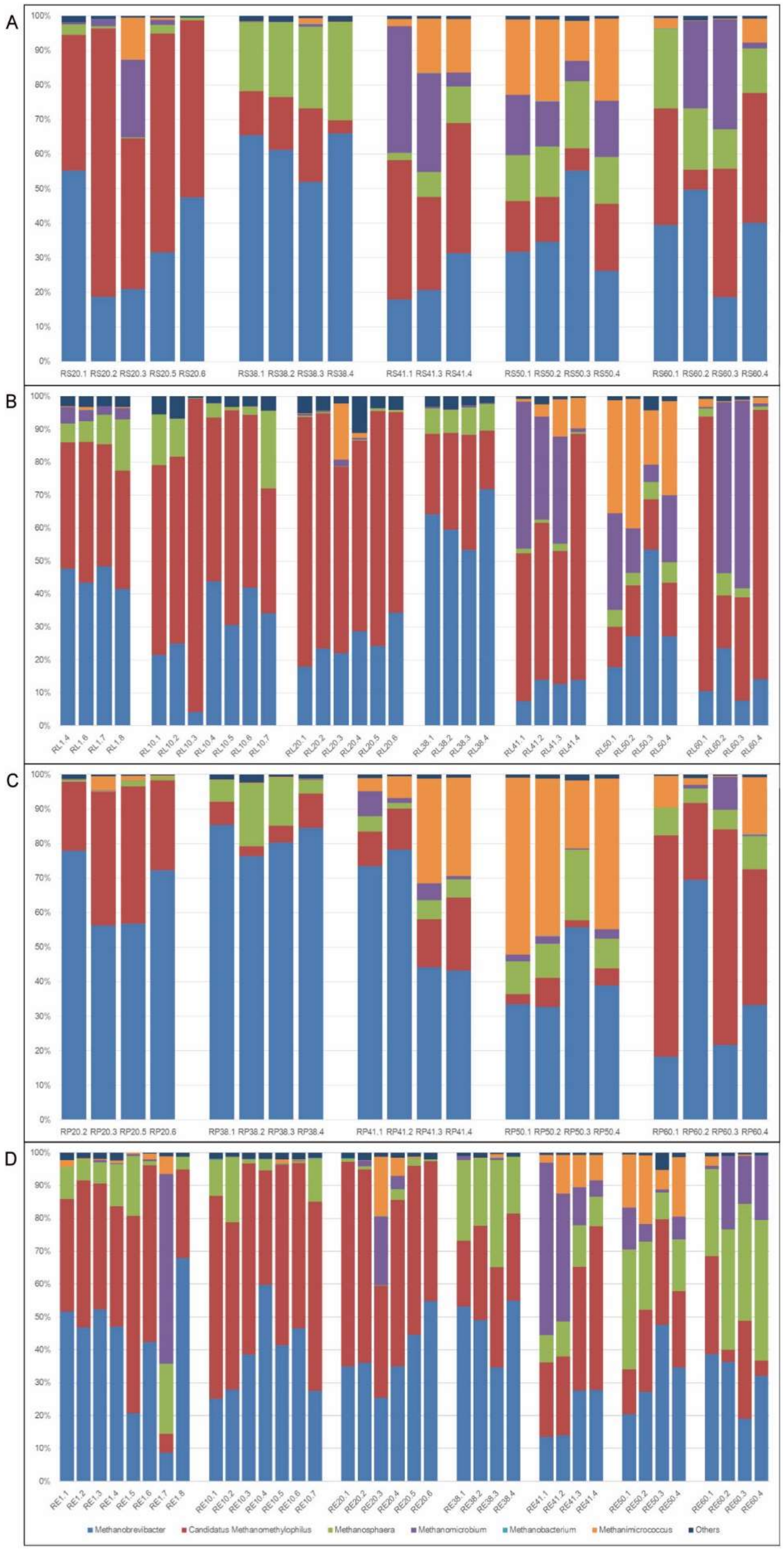

Supplementary Figure S2. Composition of methanogen communities at genus level in four fractions of control diet treatment and rhubarb supplementation treatment across individuals on 50 d (A) and 60 d (B).

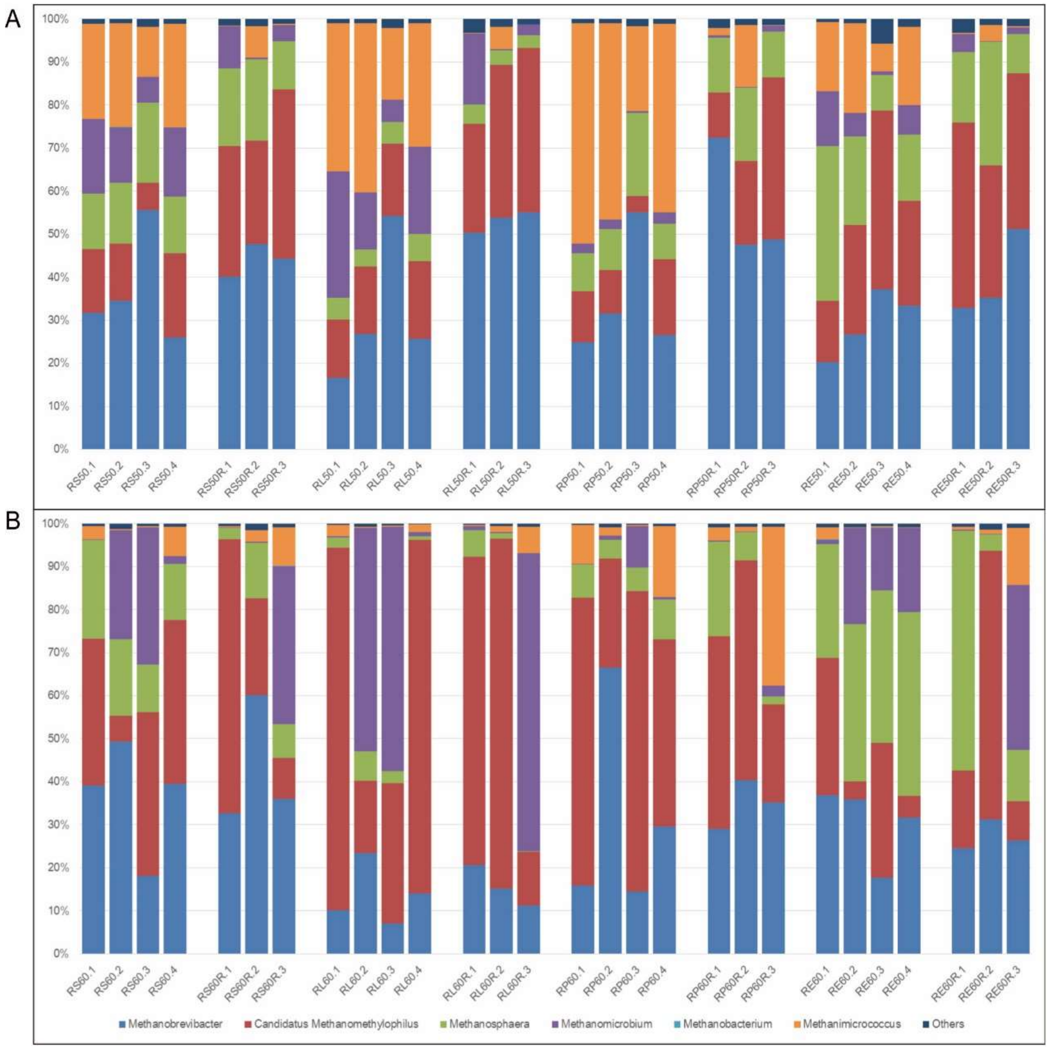

Supplementary Figure S3. Comparison of Chao 1 index of methanogen communities within different fractions on different days. The horizontal lines in each box indicate the median values, and the 75th and 25th quartile values are respectively represented by the top and bottom sides of each box.

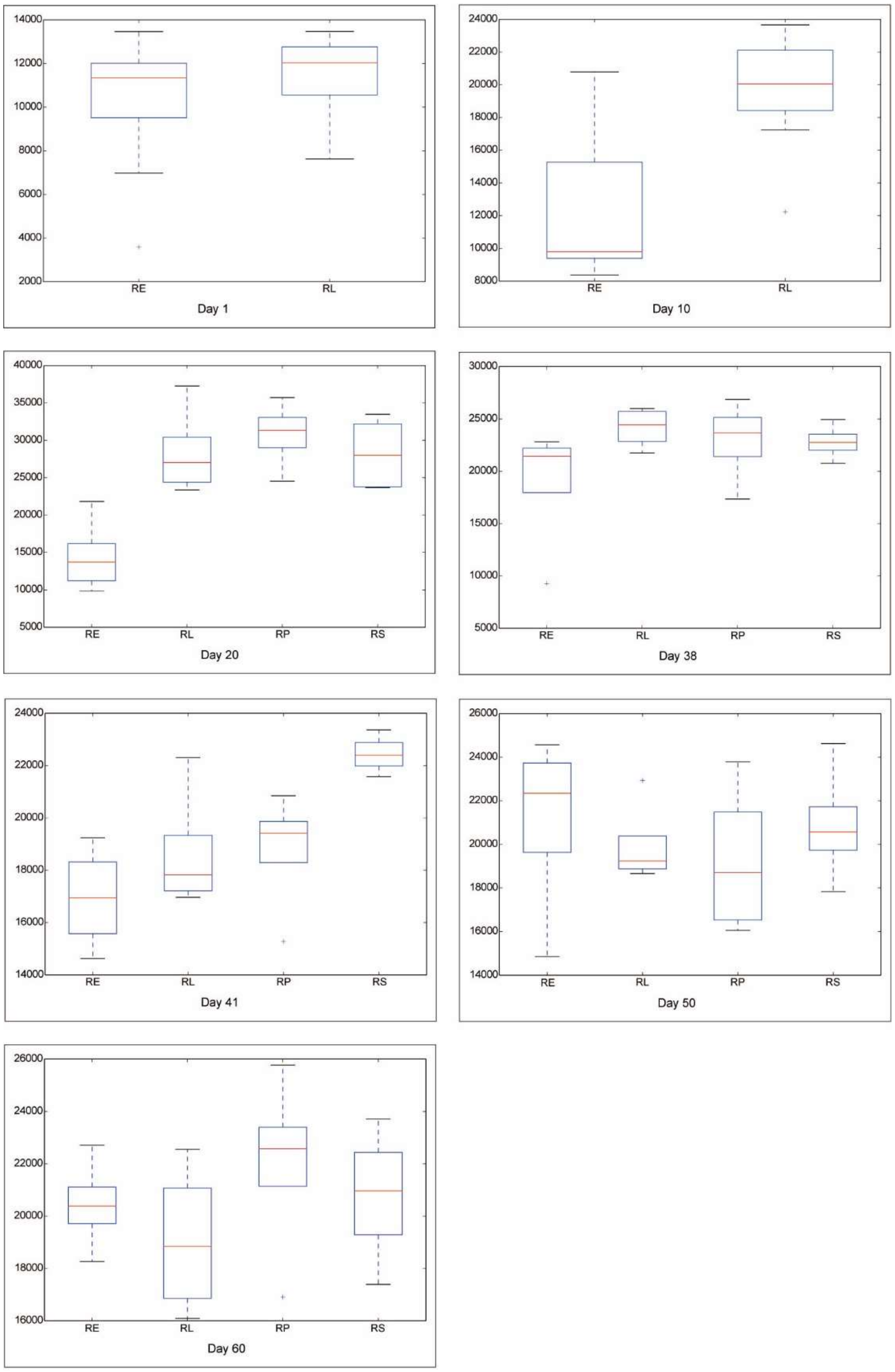

Supplementary Figure S4. Principal coordinate analysis (PCoA) of methanogen community structure using unweighted Unifrac matrix within different fractions on different days.

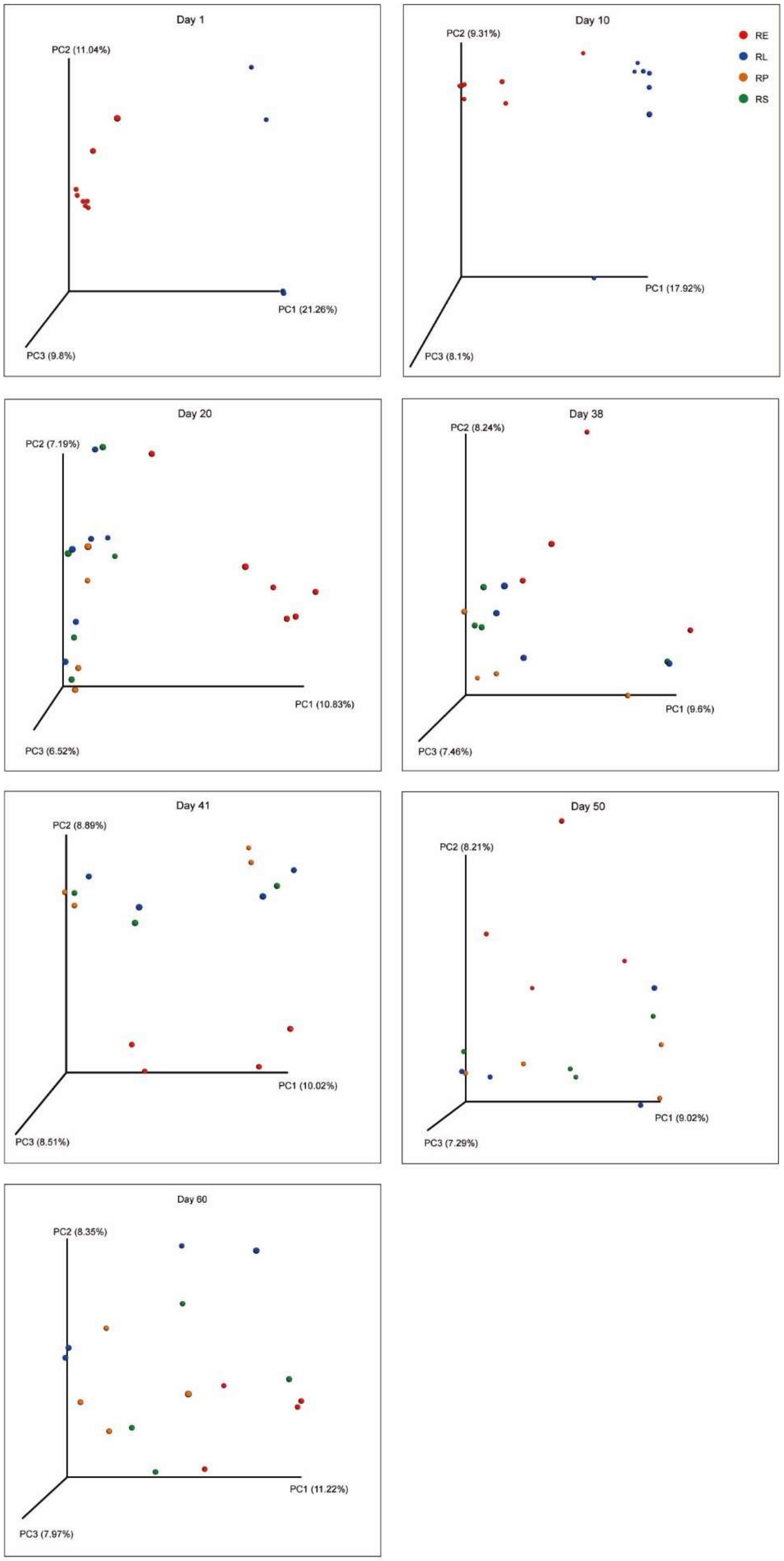

Supplementary Figure S5. Management of goats and sampling from four fractions during rumen development.

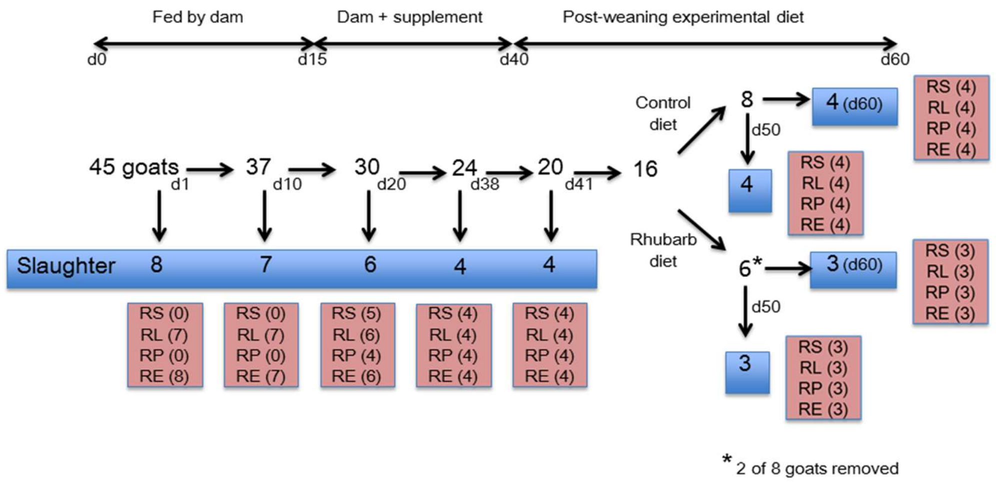

Supplementary Figure S6. Increase in body weight of black goats during rumen development.

Note: There was no significant difference (P=0.736) in body weight between the control diet treatment and the rhubarb supplementation treatment.

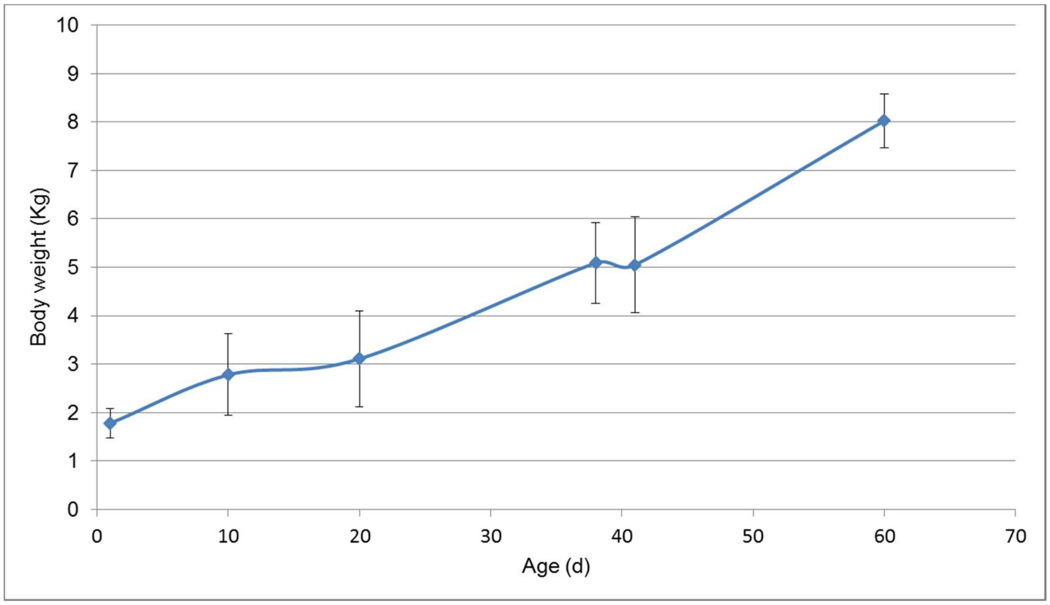

Supplement: Supplementary file 1 — Supplementary Information [file 41598_2017_500_MOESM1_ESM.pdf]
